# Sub-Clinical Effects of Chronic Noise Exposure on Vestibular System

**Published:** 2020-05-31

**Authors:** P Viola, A Scarpa, D Pisani, C Petrolo, T Aragona, L Spadera, P De Luca, FM Gioacchini, M Ralli, E Cassandro, C Cassandro, G Chiarella

**Affiliations:** 1 Unit of Audiology, Department of Experimental and Clinical Medicine, Regional Centre for Cochlear Implants and ENT Diseases, University “Magna Graecia”, Catanzaro, Italy; 2 Department of Medicine and Surgery, University of Salerno, Salerno, Italy; 3 Otolaryngology, A.O.U. Ospedali Riuniti, Ancona, Italy; 4 Otolaryngology, San Leonardo Hospital, Castellammare di Stabia, Napoli, Italy; 5 ENT Unit, Department of Clinical and Molecular Sciences, Polytechnic University of Marche, Ancona, Italy; 6 Department of Sense Organs, University Sapienza of Rome, Italy; 7 Department of Surgical Sciences, University of Turin, Turin, Italy

**Keywords:** chronic noise-induced hearing loss, acoustic trauma, vestibular damage, cVemps

## Abstract

**Aim:**

to investigate the effect of chronic noise exposure on vestibular function of subjects without clinical evidence of vestibular disorders and with documented cochlear damage from noise.

**Subjects and methods:**

25 patients with chronic noise-induced hearing loss (NIHL) and without vestibular complaints (group A) and 25 matched controls with sensorineural hearing loss without noise exposure (group B), underwent audiological and vestibular test including caloric and cervical vestibular-evoked myogenic potentials tests (cVEMPs).

**Results:**

In subjects chronically exposed to noise, similarly to that of the auditory threshold, an increase in the evocation threshold of VEMPs has been documented, statistically significant (p<0,05) and independent of the performance of the auditory threshold. p1-n1 amplitude values showed a significant difference between group A and group B. No significant difference for p1-n1 latencies between the two groups was found.

**Conclusion:**

We have documented the possibility of vestibular lesion, along with cochlear damage, related to chronic acoustic trauma.

## I. INTRODUCTION

Noise induced hearing loss (NIHL) is an insidious and cumulative disease that worsens over time. Over the last years, literature showed a large and increasing interest in NIHL^1^,^2^, while the effect of noise on vestibular system has been given less attention and remained only a secondary aspect of acoustic trauma^3^,^4^.

Currently, few clinical studies have been performed about vestibular function and noise exposure, many of these studied^5^–^7^ only complaints of vestibular symptoms and disequilibrium without instrumental examination and the results are often highly controversial^8^,^9^,^10^.

From an anatomical point of view, there are three fundamental issues for the rationale of a vestibular acoustic damage similar to NIHL: the proximity of vestibular organs to the acoustic-energy delivery system^11^,^12^ the similarity of cellular structure of cochlear and vestibular receptors and the common blood supply of cochlear and vestibular organs^4^.

Animal research showed that vestibular organs may be damaged by noise exposure just like the cochlea^13^,^14^, typically the saccule is more affected than utricle and semicircular canals^15^–^20^. It’s well known that acoustically responsive saccular afferents determine acoustic reflexes of the sternocleidomastoid (SCM) muscle and the measurement of these reflexes reflects well the saccular function^21^,^22^. So, the most useful tool to evaluate saccule’s function is cervical vestibular evoked myogenic potentials (cVEMPs)^23^,^24^

Focusing on this rationale, the aim of this study is to evaluate saccular function using cVEMPs in subjects with documented NIHL from chronic occupational noise exposure and without vestibular symptoms.

## II. MATERIALS AND METHODS

### Subjects

This retrospective study was conducted at the Audiology Unit of the University of Magna Graecia of Catanzaro, Italy, between January 2010 and May 2011, and enrolled 25 male patients (Group A) (age 25–63, mean 50,6) affected by chronic NIHL according American College of Occupational and Environmental Medicine (ACOEM) criteria^25^.

A Control group (Group B) enrolled 25 male subjects (age 25 – 69 years old, mean 47,36) affected by bilateral sensorineural hearing loss (SNHL), never exposed to occupational noise.

Patients signed a written informed consent; the procedures performed were in accordance with the standards of the ethics committee on human experimentation of the University of Catanzaro, that specifically approved this study, and with the Helsinki Declaration.

Inclusion criteria were age between 25 and 70 years old; documented exposure to occupational noise for at least 5 years; audiogram configuration and shape typical of acoustic trauma.

Exclusion criteria were other external or middle ear abnormalities, family history of otological disorders, prior or ongoing exposure to drugs possibly affecting hearing and vestibular function, retrocochlear pathology, previous ear surgery, previous or ongoing vestibular symptomatology.

### Methods

A clinical interview investigating medical and occupational history was performed. All subjects underwent a detailed audio vestibular examination as described below.

### Audiological Evaluation

The audiological evaluation included otoscopic examination, pure-tone audiometry (Amplaid 319 Amplimedica, Italy) and acoustic immittance measures (Amplaid A766 Amplimedica, Italy).

### Pure-Tone Audiometry

Hearing thresholds were measured by audiometer (Amplaid 319 Amplimedica, Italy) inside a soundproof audiometric booth. The lowest-intensity sound subjects could hear for each frequency was recorded (0.125, 0.25, 0.5, 1, 2, 4, 6, and 8 kHz for air conduction, 0.25, 0.5, 1, 2, 4, 6, and 8 kHz for bone conduction). A pure-tone average (PTA) of air conduction thresholds at 0.5, 1, 2, 4, 6 and 8 kHz was calculated for each ear separately and the mean PTA was calculated. Hearing impairment was ranked as mild, moderate, severe, or profound as follows: mild loss, 26–40 dB; moderate loss, 41–70 dB; and severe loss, 71–90 dB, profound, 91+dB^26^.

### Caloric test

Vestibular caloric test was performed according to Fitzgerald and Hallpike method^27^ and eyes movements were registered by videonystagmography (VNG Ulmer® system).

### cVEMPs

cVEMPs were assessed by active electrodes placed on the upper half of the bilateral SCM muscles, while reference and ground electrodes were placed on the suprasternal notch and forehead, respectively. Electromyographic (EMG) signals were amplified, bandpass-filtered between 10 and 200 Hz, and monitored to maintain background muscle activity at over 50 μV. Acoustic stimuli were a 500 Hz logon with rarefaction polarity and were delivered through an insert earphone starting at 130 dB SPL and reaching thresholds. An average of 100 responses was recorded for each run, with the subject sitting with the head rotated sideways towards one shoulder to activate the SCM muscle. The cVEMPs was measured by monaural acoustic stimulation with ipsilateral recording. The first positive and second negative polarities of biphasic waveform were termed waves p1 and n2, respectively. Consecutive trials were performed to confirm the reproducibility of p1 and n2 peaks following which cVEMPs reproducible responses were considered present. Conversely, cVEMPs responses were considered absent when the biphasic p1–n2 waveform was not reproducible.

## III. STATISTICAL ANALYSIS

Statistical analyses were performed using Fisher’s test and Chi-Square Test. The p-value for assessing statistical significance was an alpha of 0.05. To obtain a better statistical evaluation, we decided to consider the number of ears, so a total of 50 ears were analyzed for each Group.

## IV. RESULTS

VEMP’s threshold in both groups is showed in table 1.

The Chi-Square test showed a significant difference for threshold values between the two groups. (p<0,05). In any patients of group A it wasn’t possible to obtain an evoked response at 100 dB SPL. In most ears, threshold values were evoked at 120 dB SPL. In six ears it was not possible to evoke VEMPs at 130 dB SPL. On the contrary, in almost all patients of group B it was possible to evoke VEMPs response at 100 dB SPL.

The amplitude of p1 and n1 (p1-n1 amplitude) and their latency (p1/n1 latencies) for each group is showed in table 2.

The T-Student test found no significant difference for p1-n1 latencies between the two groups. Conversely, p1-n1 amplitude values showed a significant difference between group A and group B.

## V. DISCUSSION

From an anatomical point of view, there are three fundamental issues for the rationale of a vestibular acoustic damage similar to NIHL: the proximity of vestibular organs to the acoustic-energy delivery system, the similarity of cellular structure of cochlear and vestibular receptors and the common blood supply of cochlear and vestibular organs. On the other side the “limiting membrane” between saccule and the rest of vestibule (utricle and semicircular canals) may protect a great part of sensory cells in the labyrinth from acoustic trauma^28^. In fact, McCabe et al.^29^ showed on animal model, that a high level sound (136–150dB SPL) for 20 min affect saccule and pars inferior of cochlea despite utricle and pars superior of semicircular canals. This difference between pars superior and inferior is probably due to the presence of the membrana limitans, that acts as a barrier, causing different sensitivity of cochlear and vestibular sensory epithelia to the pathogen noxa like acoustic exposure. Embryologically the semicircular ducts arise from utricle and the cochlear duct develop from a comparative large saccule as evidenced in the lower species which can serve as an acoustic receptor. In the literature there are studies on VEMPs following acute acoustic trauma for almost all only on experimental animal, while limited experiences on chronic exposure in humans are reported. Several studies showed evidence of associated damage of cochlea and vestibular organs1^4^,^19^,^30^ and Ylikoski^19^ suggests a resemblance between type of damage in the two structures.

The aim of this study was to evaluate saccular function by cVEMPs in a sample of NIHL patients with normal caloric tests comparing to patients affected by bilateral sensorineural hearing loss never exposed to acoustic trauma. Our results showed that chronic noise exposure produces subclinical saccular damage as confirmed by elevated threshold values and reduced p1-n1 amplitude of VEMPs in group A comparing group B. No significant difference we have found between two groups for p1-n1 latencies values.

Our results are partly in accordance with other studies available in the literature. Tseng and Young^10^ noted that patients subjected to NIHL decrease order of abnormal percentages in the function of the cochlea, saccule, utricle and semicircular canals. Giorgianni et al^30^ reported that in 60 subjects with NIHL cVEMPs were abnormal or absent in 64.9%, specifically, cVEMPs were absent in 28.3%, latency was increased and peak- to-peak amplitude was reduced in 36.6%. Wang et al.^31^ (2007) reported that patients with bilateral 4-kHz notched audiogram and hearing threshold of 4 kHz > 40 dB show abnormal (absent or delayed) cVemps, indicating that the vestibular part, especially the sacculo-collic reflex pathway, has also been damaged. Same results are showed by Kumar et al.^32^: they reported that in NIHL, as the pure tone average increased, the latency of p1-n1 cVEMPs was prolonged, and peak to peak amplitude was reduced in experimental group when compared to control group (normal hearing). Abd El-Salam et al^33^ reported that cVEMPs latency was increased and cVEMPs amplitude was reduced in NIHL subgroups versus the control group consisting of 20 healthy volunteer adults with normal hearing thresholds and no history of noise exposure.

The only different data in our work with respect to those mentioned is the longer p1-n1 latencies, which was not statistically significant compared to the control group. This is probably due to the fact that in the previous studies the control group is composed by normal hearing patients, while in our study the control group includes subjects affected by sensorineural hearing loss. Furthermore, the last two Authors mentioned^32^,^33^ did not perform vestibular tests including caloric stimulation in order to exclude a canal damage and inferior vestibular nerve injury that, as it is known, may affect the sacculo-collic reflex evaluated by cVEMPs.

The results of our study confirm the starting hypothesis: the sensitivity of the saccule and some particular anatomical conditions make this portion of the posterior labyrinth susceptible to noise damage almost like the cochlear receptor. On the one hand the raising of the VEMPs thresholds in subjects chronically exposed to noise, statistically significant compared to the control group, and on the other the demonstration that it is not related to the extent of the hearing loss caused by the noise damage, leads us to believe that there is damage from chronic exposure to noise affecting the saccular receptors and that this can be documented through the study of VEMPs. Finally, the clinical expression of this vestibular deficit is likely limited by the condition of compensation for damage that has slowly arisen.

## VI. CONCLUSION

We have documented the possibility of vestibular lesion, along with cochlear damage, related to chronic acoustic trauma. The diagnosis of this condition requires the simple registration of VEMPs and can have clinical significance as well as obvious medico-legal implications. It is in fact possible to hypothesize that the asymptomatic condition may in any case fail in changed environmental conditions and potentially also constitute a danger to safety. It may be appropriate to add VEMPs to the evaluation protocol of subjects chronically exposed to noise and affected by hearing loss hearing loss. Our research, as previous studies, confirm that chronic noise exposure damages vestibular organs, particularly the saccule, and that typical electrophysiological signs are higher threshold and low p1-n1 amplitude of VEMPs.

## Figures and Tables

**Table 1:** Distribution of VEMP’s threshold values in the two groups for each ear (group A e B)

	Group A		Group B	
dB SPL	right car	left car	right car	left car

100	0	0	23	25

110	8	0	2	0

120	9	12	0	0

130	5	1	0	0

**Table 2:**
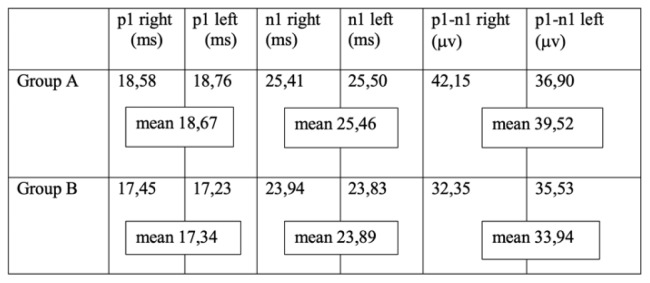
Average values of cVEMP’s p1/n1 latencies (ms) and the p1-n1 amplitude (μv) for right and left ear in each group.
